# Myocardial Effective Transverse Relaxation Time T_2_^*^ is Elevated in Hypertrophic Cardiomyopathy: A 7.0 T Magnetic Resonance Imaging Study

**DOI:** 10.1038/s41598-018-22439-x

**Published:** 2018-03-05

**Authors:** Till Huelnhagen, Min-Chi Ku, Henning Matthias Reimann, Teresa Serradas Duarte, Andreas Pohlmann, Bert Flemming, Erdmann Seeliger, Christina Eichhorn, Victor A. Ferrari, Marcel Prothmann, Jeanette Schulz-Menger, Thoralf Niendorf

**Affiliations:** 1 Berlin Ultrahigh Field Facility (B.U.F.F.), Max Delbrück Center for Molecular Medicine in the Helmholtz Association, Berlin, Germany; 2 DZHK (German Centre for Cardiovascular Research), partner site, Berlin, Germany; 3 Institute of Vegetative Physiology, Charité University Medicine, Berlin, Germany; 4 Statistical Sciences, Department of Information Technology, Max Delbrück Center for Molecular Medicine in the Helmholtz Association, Berlin, Germany; 5 Division of Cardiovascular Medicine and Penn Cardiovascular Institute, Perelman School of Medicine, University of Pennsylvania, Philadelphia, USA; 6 Working Group on Cardiovascular Magnetic Resonance, Experimental and Clinical Research Center, a joint cooperation between the Charité Medical Faculty and the Max Delbrück Center for Molecular Medicine, Berlin, Germany; 7 Experimental and Clinical Research Center, a joint cooperation between the Charité Medical Faculty and the Max Delbrück Center for Molecular Medicine, Berlin, Germany

## Abstract

Hypertrophic cardiomyopathy (HCM) is the most common genetic disease of the myocardium and bares the risk of progression to heart failure or sudden cardiac death. Identifying patients at risk remains an unmet need. Recognizing the dependence of microscopic susceptibility on tissue microstructure and on cardiac macromorphology we hypothesized that myocardial T_2_^*^ might be altered in HCM patients compared to healthy controls. To test this hypothesis, myocardial T_2_^*^-mapping was conducted at 7.0 Tesla to enhance T_2_^*^-contrast. 2D CINE T_2_^*^-mapping was performed in healthy controls and HCM patients. To ensure that T_2_^*^ is not dominated by macroscopic magnetic field inhomogeneities, volume selective B_0_ shimming was applied. T_2_^*^ changes in the interventricular septum across the cardiac cycle were analyzed together with left ventricular radius and ventricular septal wall thickness. The results show that myocardial T_2_^*^ is elevated throughout the cardiac cycle in HCM patients compared to healthy controls. A mean septal T_2_^*^ = 13.7 ± 1.1 ms (end-systole: T_2_^*^_,systole_ = 15.0 ± 2.1, end-diastole: T_2_^*^_,diastole_ = 13.4 ± 1.3 ms, T_2_^*^_,systole_/T_2_^*^_,diastole_ ratio = 1.12) was observed in healthy controls. For HCM patients a mean septal T_2_^*^ = 17.4 ± 1.4 ms (end-systole: T_2_^*^_,systole_ = 17.7 ± 1.2 ms, end-diastole: T_2_^*^_,diastole_ = 16.2 ± 2.5 ms, T_2_^*^_,systole_/T_2_^*^_,diastole_ ratio = 1.09) was found. Our preliminary results provide encouragement that assessment of T_2_^*^ and its changes across the cardiac cycle may benefit myocardial tissue characterization in HCM.

## Introduction

Hypertrophic cardiomyopathy (HCM) is the most common genetic disease of the myocardium^1^. Epidemiological studies estimated the prevalence of HCM to be about 0.2–0.5% in the general population^1,2^. The disease is characterized by myocardial hypertrophy in absence of an obvious extrinsic cause such as pressure or volume overload^3^. Patients often remain asymptomatic, but the disease can have a severe outcome in a subgroup of patients where it may cause heart failure and unexpected sudden cardiac death (SCD) in any age group. Major basic research efforts and clinical science activities are underway to better characterize HCM patient populations^4^.

Cardiovascular magnetic resonance (CMR) imaging has emerged as an indispensable tool in the diagnosis and risk stratification of HCM^3,5–7^. Vigorous research has resulted in an enormous body of literature that documents the merits of CMR and showed that the degree of hypertrophy or presence of myocardial fibrosis are associated with a poor outcome in HCM^8–11^. Notwithstanding this success, understanding the pathophysiologic mechanisms of the imaging findings and identifying patients at risk of SCD or progression to heart failure remains an unmet clinical need^4^. So far it has been extremely challenging to connect the molecular and cellular defects that characterize HCM to the level of major organ systems at which they play themselves out.

Quantitative mapping of the effective transversal relaxation time T_2_^*^ provides valuable means for myocardial tissue characterization without the need for exogenous contrast^12,13^. A growing number of reports refers to mapping T_2_^*^ in basic research and emerging clinical CMR applications^14,15^. Myocardial T_2_^*^ is commonly assumed to provide a surrogate for myocardial tissue oxygenation^14^. Yet, the factors influencing T_2_^*^ are of multiple nature^16^. Further to blood oxygenation, blood volume fraction per tissue volume, hematocrit, the oxyhemoglobin dissociation curve, main magnetic field inhomogeneities, tissue pH, tissue susceptibility, tissue iron content and tissue microstructure or micromorphology were reported to govern T_2_^* ^^13,17,18^. Cardiac macromorphology including ventricular radius and ventricular wall thickness constitutes another category of physiological parameters that orchestrate T_2_^* ^^19^.

The linear relationship between magnetic field strength and microscopic susceptibility effects renders ultrahigh field (B_0_ ≥ 7.0 T) CMR conceptually appealing for myocardial T_2_^*^ mapping^20,21^. The enhanced susceptibility effects at 7.0 Tesla (T) may be useful to lower the detection level and to extend the dynamic range of the sensitivity for monitoring T_2_^*^ changes^19^. Moving to ultrahigh magnetic fields also enables cinematic T_2_^*^ mapping in scan times feasible for breath held acquisitions^22^. Taking advantage of this gain, temporally resolved T_2_^*^ mapping at 7.0 T showed cyclic changes of myocardial T_2_^*^, demonstrated a close correlation with myocardial wall thickness and myocardial wall stress and suggested an association with alterations in myocardial blood volume fraction across the cardiac cycle^23^. These findings hold the potential to exploit T_2_^*^ mapping for non-invasive probing of myocardial (patho)physiology *in vivo*^24^.

It is established in the literature that HCM can cause alterations in the microstructure of myocardial tissue and can induce changes in cardiac macromorphology including myocardial wall thickening and reduction in left ventricular inner radius. Based on the dependence of microscopic magnetic field pertubations on such changes^25,26^ we hypothesize, that myocardial T_2_^*^ and its time course across the cardiac cycle might be altered in HCM patients compared to healthy controls and hence might provide an imaging based marker for HCM. To test this hypothesis myocardial T_2_^*^ of the intraventricular septum was examined at 7.0 Tesla using high spatio-temporally resolved, susceptibility weighted 2D CINE techniques in healthy controls and in HCM patients. This approach was paralleled by an assessment of the patterns and degree of myocardial hypertrophy.

## Methods

### Study population

Six healthy volunteers without any known history of cardiac disease (4 male, age = 50.0 ± 12.4 years (mean ± sd), BMI = 23.9 ± 2.9 kg/m^2^) and six patients with confirmed HCM (4 male, age = 52.7 ± 17.5 years (mean ± sd), BMI = 25.2 ± 1.9 kg/m^2^) were included in the study (Table 1) after due approval by the local ethical committee (ethics committee name: *Ethikkommission der Charité, Ethikkommission 1 am Campus Charité-Mitte*, Berlin, Germany; registration number DE/CA73/5550/09, Landesamt für Arbeitsschutz, Gesundheitsschutz und technische Sicherheit, Berlin, Germany). *In vivo* studies were approved by the local ethical committee. The diagnosis of HCM was based on clinical parameters including echocardiography^3^. Informed written consent was obtained from each volunteer prior to the study in compliance with the local institutional review board guidelines. All experiments were performed in accordance with the Declaration of Helsinki and the local institutional review board guidelines.

**Table 1:** Subject characteristics.

Parameter	HCM Patients	Healthy Controls	*P* Value
n	6	6	
Sex (male/female)	4/2	4/2	
Age, y	52.7 ± 17.5	50 ± 12.4	0.77
Height, cm	170 ± 10	172 ± 8	0.71
Weight, kg	73.12 ± 9.6	71.5 ± 14.1	0.82
BMI, kg/m^2^	25.19 ± 1.9	23.9 ± 2.9	0.39
BSA, m^2^	1.84 ± 0.18	1.84 ± 0.21	0.97
Systolic blood pressure, mmHG	142.3 ± 22.8	135.5 ± 14.9	0.56
Diastolic blood pressure, mmHG	85.8 ± 17.5	86.3 ± 13.6	0.96
Heart rate, min^−1^	65.17 ± 10.83	71.7 ± 9.8	0.30
Mean end-systolic septal wall thickness, mm	16.6 ± 1.8	9.8 ± 1.4	0.001*
Mean end-diastolic septal wall thickness, mm	13.0 ± 3.1	6.2 ± 1.2	0.002*
Mean T_2_^*^ averaged across all phases, ms	17.4 ± 1.4	13.7 ± 1.1	0.001*
Mean end-systolic septal T_2_^*^, ms	17.7 ± 1.2	15.0 ± 2.1	0.025*
Mean end-diastolic septal T_2_^*^, ms	16.2 ± 2.5	13.4 ± 1.3	0.039*
LVEDV, ml	128.8 ± 33.3	121.02 ± 21.88	0.64
LVESV, ml	51.3 ± 20.0	49.35 ± 12.96	0.85
LVEF	60.9 ± 8.5	59.3 ± 6.3	0.47
LV mass, g	168.9 ± 68.0	93.1 ± 17.0	0.041*
Presence of late gadolinium enhancement	6/6	—	

Values are given as mean ± standard deviation. The *P* value stems from a student’s t-test except for LVEF where it stems from a Mann-Whitney u-test. BMI, body mass index; BSA, body surface area; *P < 0.05.

### Late Gadolinium enhancement imaging

All HCM patients included in the study had previously undergone a clinical MRI exam including LGE imaging for detection of myocardial fibrosis^27^. For this purpose a 3.0 T MR system (Magnetom Verio, Siemens, Erlangen, Germany) was employed using a 32-channel RF receive array. LGE images were acquired 10–15 minutes after application of gadobutrol (0.2 mmol/kg body weight) using a FLASH inversion recovery gradient echo technique to detect fibrosis. Imaging parameters were: TE = 5.4 ms, TR = 10.5 ms, flip angle 30°, spatial resolution (1.5 × 1.5 × 6.0) mm^3^. LGE imaging at 3.0 T was performed for five of the six patients. For the sixth patient LGE imaging data were available from a 1.5 T system (Magnetom Avanto, Siemens, Erlangen, Germany). The slice planning during the current study was based on the previous clinical exams and carefully adjusted to achieve the same slice positioning.

### B_0_ shimming

To minimize the influence of macroscopic magnetic field inhomogeneities on T_2_^*^ and to ensure that T_2_^*^ is not dominated by macroscopic magnetic field inhomogeneities but rather governed by microscopic susceptibility effects, volume selective B_0_ shimming was carefully carried out prior to T_2_^*^ mapping^23^. A B_0_ field map was acquired in a single breath hold in end-diastole using an axial stack of slices covering the entire heart. A cardiac triggered multi-echo gradient-echo technique (TEs = 2.04 ms and 4.08 ms, TR = 5.4 ms, spatial resolution (4.2 × 4.2 × 8.0) mm^3^, 18 slices) was employed. Based on this field map second order shimming was applied for a shim volume accommodating a four chamber view and a mid-ventricular short axis view of the heart^23^. This shimming approach yielded magnetic field homogeneity in the heart comparable to what has been reported at 3.0 T^22^.

### CINE imaging and T_2_^*^ weighted image acquisition at 7.0 T

Experiments were performed in a 7.0 T whole body MR system (Magnetom, Siemens, Erlangen, Germany). A 16 channel transceiver RF coil array was used^28^. An MR stethoscope (EasyACT, MRI.TOOLS GmbH, Berlin, Germany)^29^ and pulse oximetry were employed for cardiac triggering and gating.

**Figure 1:**
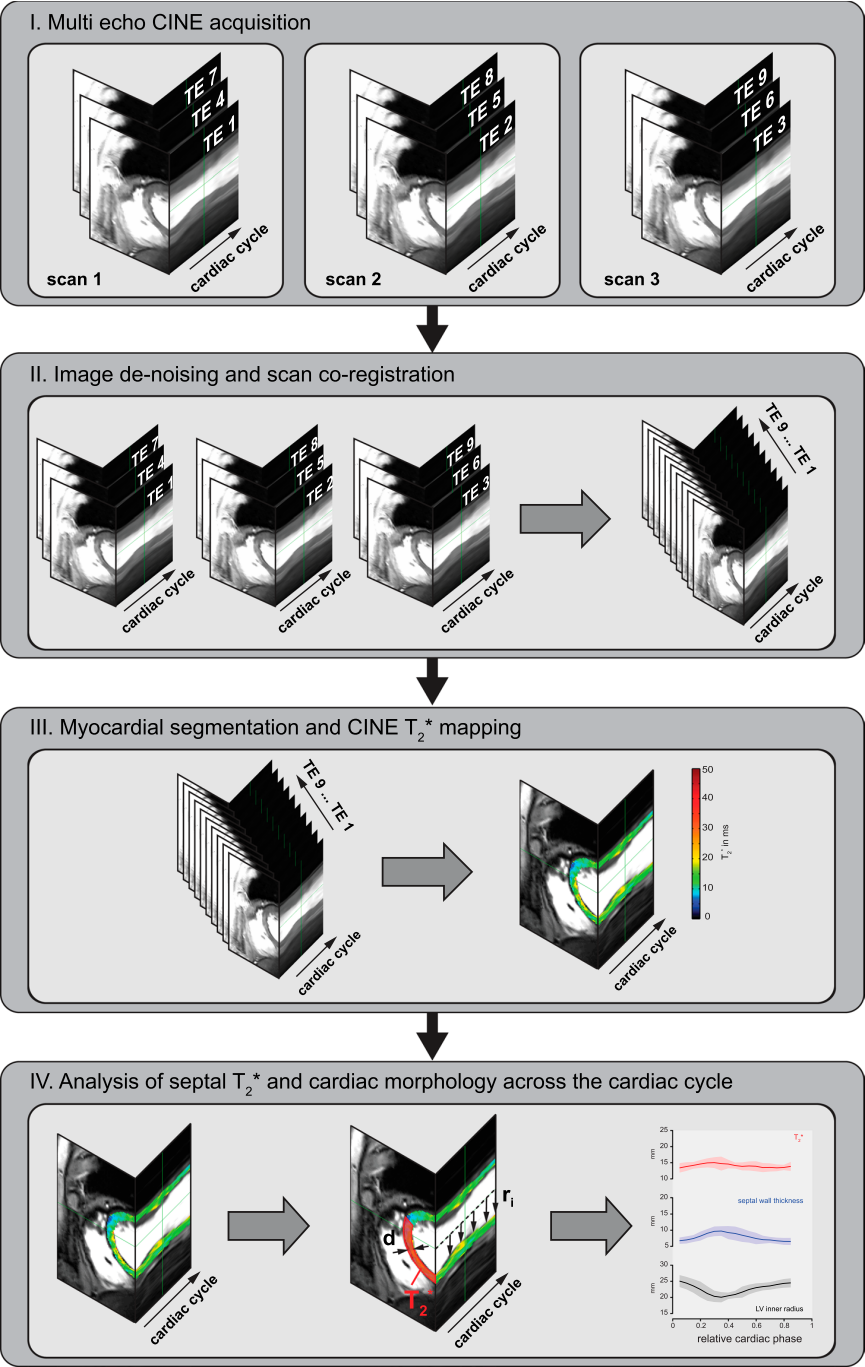
Image acquisition, post processing and data analysis pipeline. (**I**) Multi echo CINE image series are acquired, (**II**) de-noised, co-registered and combined. (**III**) Myocardium is segmented in 2.5° wide radial sections covering the whole myocardium and T_2_^*^ mapping is performed. (**IV**) Finally basic cardiac morphology and septal T_2_^*^ are being analyzed.

High spatial resolution CINE T_2_^*^ mapping was performed with a segmented multi-shot multi breath hold gradient echo technique^22^ using: TE = 2.04–10.20 ms, ΔTE = 1.02 ms, TR = 12.16 ms, spatial resolution (1.1 × 1.1 × 4.0) mm^3^, flip angle 20°, GRAPPA acceleration factor 4. T_2_^*^ weighted CINE acquisitions were split in three sub-acquisitions, each acquired in one breath-hold^22^ (Fig. 1I). High blood-myocardium contrast 2D CINE FLASH acquisitions (spatial resolution (1.4 × 1.4 × 4.0) mm^3^, flip angle 32°, TE = 2.67 ms, TR = 5.66 ms, GRAPPA acceleration factor 2) were used as anatomic reference. Mid-ventricular short axis views were acquired.

### Data processing

Data processing (Fig. 1) was performed offline using MATLAB (The Mathworks, Natick, MA, USA) routines. Prior to T_2_^*^ fitting, all T_2_^*^ sensitized images were de-noised using a spatially adaptive non-local means (SANLM) filter^30,31^ (VBM8 toolbox (http://dbm.neuro.uni-jena.de/vbm8/)). SANLM filtering decreased the estimated fit standard deviation of T_2_^*^ in the myocardium by approximately 32% in healthy volunteers and 46% in patients in comparison to fits from unfiltered images. No artifacts due to filtering were observed in the fitted maps^23,31^. After de-noising, the T_2_^*^ weighted CINE images from the three breath holds were co-registered employing a non-rigid registration provided by the MIRT MATLAB toolbox (https://sites.google.com/site/myronenko/research/mirt). Images of the three registered scans were combined to form multi-echo series covering nine echoes with increasing T_2_^*^ weighting (Fig. 1II). Subsequently, non-linear T_2_^*^ fitting was performed using the MATLAB trust region algorithm in combination with a mono-exponential signal decay model (Fig. 1III). Goodness-of-fit was evaluated by *R*^2^. Additionally the fit standard deviation (T_2_^*^-STD) was estimated^32^. Voxels with decreased fit quality (*R*^2^ < 0.7 or T_2_^*^-STD > 3 ms) or unnaturally high/low T_2_^*^ (T_2_^*^ ≤ 1 or T_2_^*^ ≥ 50 ms) were considered unreliable and excluded from further analysis.

### Data analysis

For each subject the left ventricular (LV) myocardium was manually segmented for all cardiac phases. LV wall thickness and inner radius were calculated for 2.5° wide radial sections covering the whole myocardium. This procedure was executed for all cardiac phases resulting in a total sample size of 18720 sections for the healthy controls and in 17712 sections for the HCM patients. Median T_2_^*^ and mean wall thickness were calculated for each cardiac phase to allow assessment of temporal changes. For this purpose only the anteroseptal and inferoseptal segments^33^ (6326 septal sections in controls and 5904 sections in HCM patients) were considered, because T_2_^*^ measurements have been shown to be most reliable in the ventricular septum^20^ (Fig. 1IV). Mean inner LV radius was calculated per phase by averaging over all sections. The averaged values of T_2_^*^, wall thickness and LV radius per cardiac phase were determined for all subjects. For calculation of group averages cardiac cycle duration was normalized and the number of phases was unified using linear interpolation. The overall distribution of septal T_2_^*^ was analyzed using the histogram of the relative frequencies of T_2_^*^ for all septal voxels in all subjects within the respective group. Evaluation of LV-morphology was described recently^27^.

### Statistical analysis

Statistical analysis was performed using R^34^ and MATLAB. Continuous data are expressed as mean ± SD. Group differences were analyzed for significance using a student’s t-test for normally distributed data and a Mann-Whitney u-test otherwise. Normal distribution was verified using a Shapiro-Wilk test. P values of *P* < 0.05 were considered significant.

### Availability of data

The datasets used and/or analysed during the current study are available from the corresponding author on reasonable request.

## Results

### Assessment of cardiac morphology

All scans could be performed without any complication. Mean examination time for the assessment of cardiac morphology was 22 ± 7 minutes in healthy volunteers and 21 ± 2 minutes in patients. Ample blood myocardium contrast with a contrast-to-noise-ratio of about 55, image sharpness and signal uniformity across the heart were achieved for all subjects. Mean septal wall thickness (SWT) averaged over all subjects for all cardiac phases was found to be 7.3 ± 1.2 mm in healthy controls and 14.1 ± 2.5 mm in HCM patients. For end-systole mean SWT was 9.8 ± 1.4 mm in healthy controls compared to 16.6 ± 1.8 mm in HCM patients (Table 1). LV analysis confirmed the substantial difference in LV-mass between healthy volunteers (93 ± 17)g and HCM patients (169 ± 68)g (*P* = 0.04). No significant differences in left ventricular end-diastolic volume (LVEDV) (*P* = 0.64), left ventricular end-systolic volume (LVESV) (*P* = 0.85) and left ventricular ejection fraction (LVEF) (*P* = 0.47) were found.

### CINE T_2_^*^ mapping

All volunteers and patients tolerated the breath-hold 2D CINE T_2_^*^ mapping acquisitions (mean examination: 1:28 ± 0:19 minutes in healthy volunteers and 1:26 ± 0:13 minutes in patients). B_0_ shimming resulted in macroscopic field dispersions of ΔB_0_ < 3 Hz per mm (in-plane) and ΔB_0_ < 1 Hz per mm (through-plane) in the ventricular septum which is similar to what has been previously reported for cardiac MRI at 7.0 T and 3.0 T indicating, that T_2_^*^ was not dominated by macroscopic magnetic field variations^22,23^.

**Figure 2:**
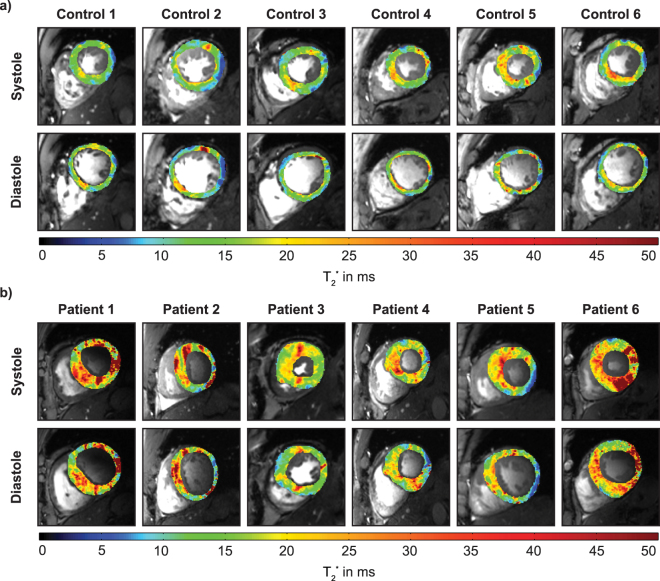
Mid ventricular short axis view of systolic and diastolic myocardial T_2_^*^ maps of healthy controls (top) and HCM patients (bottom) derived from CINE T_2_^*^ mapping superimposed to FLASH CINE images. Spatial resolution = (1.0 × 1.0 × 4.0)mm^3^. T_2_^*^ differences between systole and diastole can be observed. Distinct regions of increased T_2_^*^ can be identified in the patients.

Averaging T_2_^*^ over all healthy controls for all cardiac phases (6323 septal sections) revealed a mean T_2_^*^ = 13.7 ± 1.1 ms (Table 1). Figure 2 surveys LV T_2_^*^ maps obtained for all healthy volunteers during systole and diastole. For this cohort a mean septal T_2_^*^ = 15.0 ± 2.1 ms was observed for end-systole. For end-diastole mean septal T_2_^*^ = 13.4 ± 1.3 ms was determined. The T_2_^*^_,systole_/T_2_^*^_,diastole_ ratio was 1.12 for healthy subjects. The mean range (max – min) of T_2_^*^ over the cardiac cycle was 4.0 ± 1.2 ms which is significantly higher (*P* < 0.01) than the 0.7 ± 0.4 ms T_2_^*^ change attributed to the periodic macroscopic B_0_ variation^23^.

**Figure 3:**
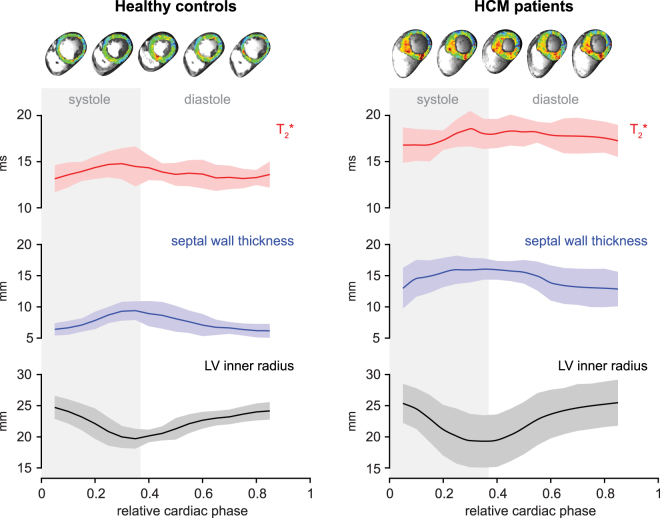
Temporal changes of ventricular septal T_2_^*^, wall thickness and inner left ventricular radius plotted over the cardiac cycle in healthy volunteers and HCM patients. Group analysis for six healthy volunteers and six patients. Shaded regions indicate SEM. Septal T_2_^*^ changes periodically over the cardiac cycle. Septal T_2_^*^ is significantly increased in HCM patients.

In comparison, averaging T_2_^*^ over all HCM patients and for all cardiac phases (5904 septal sections) revealed mean septal T_2_^*^ = 17.4 ± 1.4 ms (Table 1, Fig. 3) which was significantly higher (P < 0.001) than in healthy controls. For patients mean septal T_2_^*^ was 17.7 ± 1.2 ms at end-systole and 16.2 ± 2.5 ms at end-diastole (Fig. 2). The T_2_^*^_,systole_/T_2_^*^_,diastole_ ratio was 1.09. No significant difference was found for the mean range (max – min) of T_2_^*^ over the cardiac cycle compared to controls (T_2_^*^_(max-min)_ = 3.8 ± 1.2 ms).

**Figure 4:**
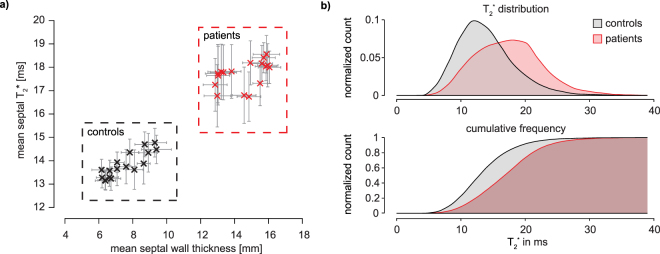
(**a**) Scatter plot of septal wall thickness and T_2_^*^ in HCM patients and healthy controls at 7.0 T. Each marker corresponds to one cardiac phase. Errorbars indicate SEM. Two clusters for patients and for volunteers can clearly be separated using mean septal T_2_^*^ and wall thickness. (**b**) Histogram (top) and cumulative frequency plot (bottom) of T_2_^*^ in the mid ventricular septum. Combined data from six healthy volunteers and six HCM patients for all cardiac phases. A clear shift toward higher T_2_^*^ can be observed in the HCM patients.

Figure 3 highlights the time course of mean septal wall thickness, median septal T_2_^*^ and mean inner ventricular radius over the cardiac cycle for HCM patients and healthy controls. Cyclic T_2_^*^ changes across the cardiac cycle were found. A T_2_^*^ increase during systole which is paralleled by an increase in the SWT and a decrease in the left-ventricular radius was observed for healthy controls and HCM patients. Also a decrease of T_2_^*^ during diastole was noted for healty controls and for HCM patients. The overall shape of the curves was similar for patients and controls, with the the diastolic T_2_^*^ decrease being less pronounced in patients. Plotting mean septal T_2_^*^ against mean septal wall thickness revealed two clearly separable clusters for HCM patients and normal controls (Fig. 4a). Analysis of the overall distribution of T_2_^*^ across all septal sections and all cardiac phases showed a significant increase of T_2_^*^ in patients compared to healthy controls (Fig. 4b).

## Discussion

This study sought to test the hypothesis, that myocardial T_2_^*^ and its time course across the cardiac cycle are altered in HCM patients compared to healthy controls. The main finding of this study is that septal T_2_^*^ is significantly elevated in HCM patients versus healthy controls. Cyclic variations of T_2_^*^ across the cardiac with T_2_^*^ increasing in systole and decreasing in diastole were observed in both, healthy controls and HCM patients with similar ratios of T_2_^*^_,systole_/T_2_^*^_,diastole_ of about 1.1. This is in line with previous reports on healthy volunteers^23^. These novel insights advance the capabilities of MR imaging based myocardial tissue characterization at a level of non-invasive interrogation not previously available in humans.

Following previous reports suggesting myocardial blood oxygenation level dependent contrast or describing T_2_^*^ as a surrogate for tissue oxygenation would lead to the conclusion that T_2_^*^ prolongation in HCM can be attributed to an increase in oxygen supply in hypertrophic versus healthy myocardium. This interpretation does not match the present knowledge though. Previous studies demonstrated that T_2_^*^ strongly depends not only on blood oxygenation but also on the tissue blood volume fraction^16,23,35^. Cyclic changes in myocardial tissue blood volume fraction which are a known physiologic phenomenon related to the contraction and relaxation of the cardiac muscle^36^ have been suggested as the main cause behind perodic variations of myocardial T_2_^*^ across the cardiac cycle in healthy volunteers^23^. It is fair to assume, that the periodic changes of T_2_^*^ observed in the HCM patient group are caused by the same mechanism. But how can the overall T_2_^*^ increase in the patient cohort be explained? Microvascular dysfunction and subsequent ischemia are a known feature in HCM^37^ and have been reported to scale with greater degrees of hypertrophy and the presence of fibrosis^38,39^. Along this line Johansson *et al*. described a 33% decrease in myocardial capillary density in HCM^40^. A reduction of vascularity in presence of scar tissue, which is also found in HCM patients, has been shown by histologic studies^41,42^. These conditions result in a reduced myocardial blood volume fraction in HCM and consequently decrease the impact of the deoxygenated hemoglobin on T_2_^*^, which could explain the observed T_2_^*^ prolongation in hypertrophic regions versus normal or remote myocardium.

An association between microvascular dysfunction and late gadolinium enhancement (LGE) has been described in HCM in the literature^38,43^. Chiribiri *et al*. reported a coincidence of mid-myocardial LGE with reduced resting state perfusion and greater degrees of hypertrophy^44^. LGE was also present in all HCM patients in the current study. This finding supports the hypothesis that the observed T_2_^*^ increase might be related to microvascular dysfunction. Reduced myocardial blood flow as observed in microvascular dysfunction and subsequent ischemia has been associated with an unfavorable outcome in HCM and suggested as a strong predictor of clinical deterioration and death^37,39^. This hints that myocardial T_2_^*^ mapping might provide an element of risk stratification in HCM. Arguably, increased myocardial T_2_ often coinciding with LGE has been described in HCM and was associated with inflammation and edema as well as ischemia and fibrosis^45,46^. A T_2_ increase would also result in elevated T_2_^*^, providing a further explanation for the observed results. Admittedly these interpretations cannot be ultimately proven at this point. Further investigations including animal models of HCM are required to better understand the relationship of MR parameter changes and underlying microstructural and pathophysiologic mechanisms which cannot be definitely verified *in vivo*. Yet, this study provides new insights and directions for further investigations which will help to link pathological processes to MR findings also in humans.

It is no secret that the increase of magnetic susceptibility effects at higher fields not only affects microscopic susceptibility changes of (patho)physiological origin but can also result in stronger macroscopic field distortions e.g. at air-tissue interfaces. This is often a concern about T_2_^*^ mapping at high and ultrahigh fields. While careful B_0_ shimming is certainly of high importance for this, in fact, dedicated shimming techniques have been shown to provide B_0_ field uniformities in the heart at 7.0 T similar to what has been described at 3.0 T^22,23^. Further to this, an often overlooked benefit of performing T_2_^*^ mapping at higher fields is the possiblity to invest the signal gain due to the higher net magnetization to realize smaller voxel sizes. This effectively reduces intravoxel dephasing due to macroscopic field gradients and their influence on T_2_^*^ and also decreases partial volume effects. The voxel size used for T_2_^*^ mapping in this study of (1.1 × 1.1 × 4.0) mm^3^ is multiple times smaller than voxel sizes commonly used for T_2_^*^ mapping in at 1.5 T ot 3.0 T which further helps to reduce the impact of macroscopic field inhomogeneities^22^.

While we recognize a limitation due to the limited number of healthy subjects and patients studied, we believe this feasibility study to be an essential precursor to a larger 7.0 T study involving healthy and HCM patient cohorts. Such a study is needed to further elucidate the underlying causes for the reported T_2_^*^ increase and further analyze the temporal behavior of T_2_^*^ over the cardiac cycle in health and in the presence of pathology.

We are only beginning to understand the meaning of the dynamics of T_2_^*^ across the cardiac cycle, but the technique holds the promise to enable further insights into myocardial (patho)physiology *in vivo*. Admittedly, the continuous motion of the myocardium currently limits temporal T_2_^*^ assessment to larger regions of interest like the septum which have to be defined for each cardiac phase. Application of motion compensation techniques to CINE T_2_^*^ mapping data would enable us to derive information on T_2_^*^ dynamics at single voxel level. Parameters like T_2_^*^ temporal standard deviation, slope, time to peak, relative T_2_^*^ change, etc. could be derived from such times series and converted to quantitative maps that may help to highlight areas of change and pathology. This approach provides an entirely new set of potential (patho)physiological imaging paramters but requires further efforts to be implemented and evaluated.

## Conclusions

Myocardial T_2_^*^ is elevated throughout the cardiac cycle in HCM patients compared to healthy controls at 7.0 T. A reduction in tissue blood volume fraction in the hypertrophied myocardium and a T_2_ increase related to inflammatory processes were suggested as potential causes for this finding. These factors have been associated with a higher risk for a poor outcome of HCM patients. Our preliminary results provide encouragement, that assessment of T_2_^*^ and its changes across the cardiac cycle may benefit myocardial tissue characterization in hypertrophic cardiomyopathy.
